# Dietary carbohydrates regulate intestinal colonization and dissemination of *Klebsiella pneumoniae*

**DOI:** 10.1172/JCI174726

**Published:** 2024-03-21

**Authors:** Aaron L. Hecht, Lisa C. Harling, Elliot S. Friedman, Ceylan Tanes, Junhee Lee, Jenni Firrman, Fuhua Hao, Vincent Tu, LinShu Liu, Andrew D. Patterson, Kyle Bittinger, Mark Goulian, Gary D. Wu

**Affiliations:** 1 Division of Gastroenterology and Hepatology, Hospital of the University of Pennsylvania, Philadelphia, Pennsylvania, USA.; 2 Division of Gastroenterology, Hepatology, and Nutrition, The Children’s Hospital of Philadelphia, Philadelphia, Pennsylvania, USA.; 3 Dairy and Functional Foods Research Unit, Eastern Regional Research Center, Agricultural Research Service, US Department of Agriculture, Wyndmoor, Pennsylvania, USA.; 4 Department of Veterinary and Biomedical Sciences, Pennsylvania State University, University Park, Pennsylvania, USA.; 5 Department of Biology, University of Pennsylvania, Philadelphia, Pennsylvania, USA.

**Keywords:** Gastroenterology, Bacterial infections, Carbohydrate metabolism, Inflammatory bowel disease

## Abstract

Bacterial translocation from the gut microbiota is a source of sepsis in susceptible patients. Previous work suggests that overgrowth of gut pathobionts, including *Klebsiella pneumoniae*, increases the risk of disseminated infection. Our data from a human dietary intervention study found that, in the absence of fiber, *K*. *pneumoniae* bloomed during microbiota recovery from antibiotic treatment. We thus hypothesized that dietary nutrients directly support or suppress colonization of this gut pathobiont in the microbiota. Consistent with our study in humans, complex carbohydrates in dietary fiber suppressed the colonization of *K*. *pneumoniae* and allowed for recovery of competing commensals in mouse models. In contrast, through ex vivo and in vivo modeling, we identified simple carbohydrates as a limiting resource for *K*. *pneumoniae* in the gut. As proof of principle, supplementation with lactulose, a nonabsorbed simple carbohydrate and an FDA-approved therapy, increased colonization of *K*. *pneumoniae*. Disruption of the intestinal epithelium led to dissemination of *K*. *pneumoniae* into the bloodstream and liver, which was prevented by dietary fiber. Our results show that dietary simple and complex carbohydrates were critical not only in the regulation of pathobiont colonization but also disseminated infection, suggesting that targeted dietary interventions may offer a preventative strategy in high-risk patients.

## Abstract 2

Klebsiella pneumonia dissemination is regulated by dietary carbohydrates that determine the abundance of this pathobiont in the human and murine intestine.

## Graphical abstract

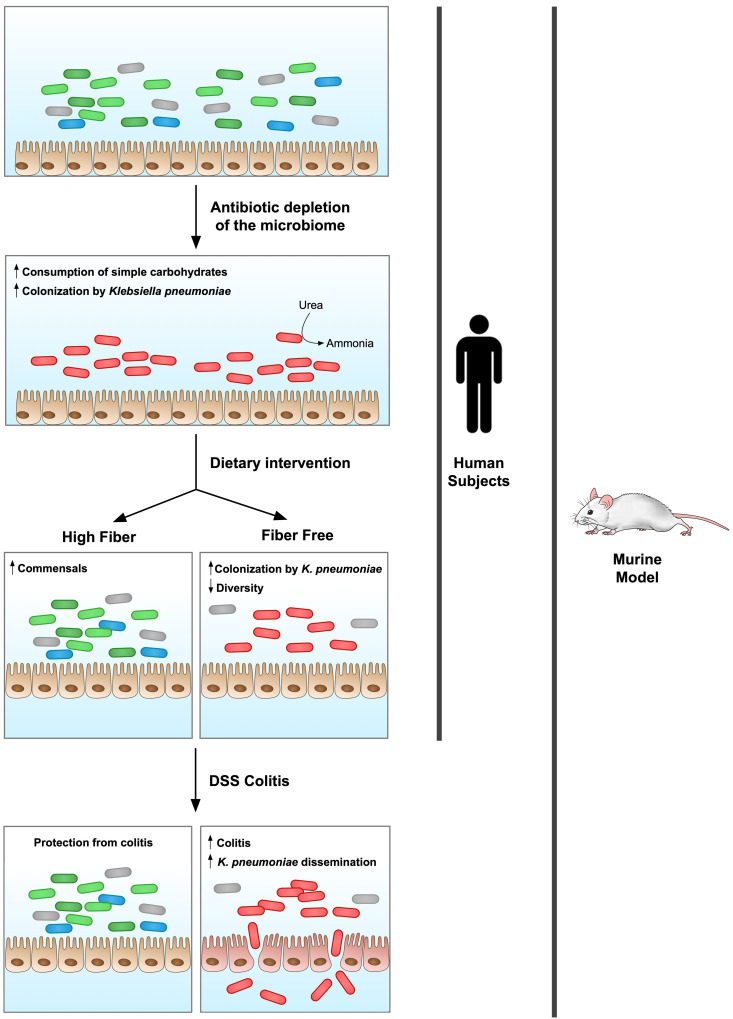

## Introduction

Bacterial translocation from the gut microbiota is a significant source of sepsis in susceptible patients, including those with cirrhosis or acute myeloid leukemia or who are admitted to the intensive care unit (1–5). Dysbiosis, the altered microbiome associated with certain chronic conditions, is thought to perpetuate sepsis through selection for opportunistic pathogens and dysregulation of the immune response (6–11). This is often characterized by a reduction of community diversity and the outgrowth of *Enterobacteriaceae*, including *Escherichia coli* and *Klebsiella pneumoniae* (6, 8, 12–14). These organisms are commonly recovered in disseminated infections, including bacteremia and urinary tract infection, suggesting a connection with gut microbiota composition (15–18). Additionally, particular strains of *E*. *coli* and *K*. *pneumoniae* have been isolated from patients with inflammatory bowel disease (IBD) and cause more severe illness in animal models (19, 20). Therefore, identification of mechanisms to increase colonization resistance against these opportunistic pathogens is essential.

Diet is a modifiable factor that is known to alter microbiota composition. Specified diets including the elimination diet and the Mediterranean diet have shown some clinical efficacy in the treatment of IBD, with improvement in the dysbiotic signature (21, 22), demonstrating that nutritional components have the potential to affect disease outcomes. The human gut microbiome response to diet is personalized, as it is dependent on the preexisting microbial population and dietary macronutrients (23–25). These macronutrients can directly affect the physiology of the resident microbiota by providing a substrate for replication to organisms capable of utilizing them (26–28).

Two essential elements for growth of all bacterial species are carbon and nitrogen. Previous work suggests that nitrogen is a limiting resource for growth of organisms in the gut microbiome (29, 30). Nitrogen is essential for anabolic processes including de novo synthesis of nucleotides and amino acids. Primary sources for nitrogen in the microbiota are dietary peptides, host-secreted digestive enzymes, glycoproteins from mucus, and the host-produced nitrogenous waste product urea, for which microbes have differing preferences (29, 31). This can in turn affect host physiology, in which intact microbiota increase the daily requirement for host protein intake (32). Carbon sources are also critical for bacterial survival; in addition to providing carbon for anabolic pathways, these molecules serve as a primary source of ATP. Dietary simple carbohydrates and starches are predominantly metabolized and absorbed by the host in the small intestine, leaving low concentrations available to the microbiota in the colon. Nondigestible complex carbohydrates, known as fiber, fall into the category of microbiota-accessible carbohydrates (MACs). The paucity of simple carbohydrates in the colon favors organisms capable of digesting dietary fibers through glycoside hydrolases (GHs) (26, 27), which have a significant effect on the microbiome in mouse and human studies (27, 33). While *Bacteroidota* and *Bacillota* can ferment these complex carbohydrates (28), very little attention has been given to the effect of dietary MACs on *Enterobacteriaceae*, as their genomes have a very low representation of genes encoding GHs necessary for their utilization (34).

We hypothesized that dietary nutrients affect colonization of *Enterobacteriaceae*. Through bacterial culture, ex vivo studies, and mouse modeling, we dissected the resource limitation of *K*. *pneumoniae*. Contrary to the commonly believed importance of nitrogen availability in the composition of the gut microbiota, we provide evidence that *Enterobacteriaceae* are not nitrogen limited but rather constrained by simple carbohydrate availability. Despite the growth-promoting effects of simple carbohydrates on *Enterobacteriaceae*, we demonstrate in both a human dietary intervention study and mouse modeling that complex carbohydrates in fiber suppress the colonization of *K*. *pneumoniae* and support the recovery of competitor commensals in the microbiota. Disruption of the intestinal epithelium induced systemic dissemination of this human pathogen, which was ameliorated by supplementation with dietary fiber. Our studies identify dietary factors critical for the perpetuation and resolution of dysbiosis in the microbiome and resulting systemic disease.

## Results

### A fiber-free diet increases the colonization of K. pneumoniae in a human study.

**Figure 1:**
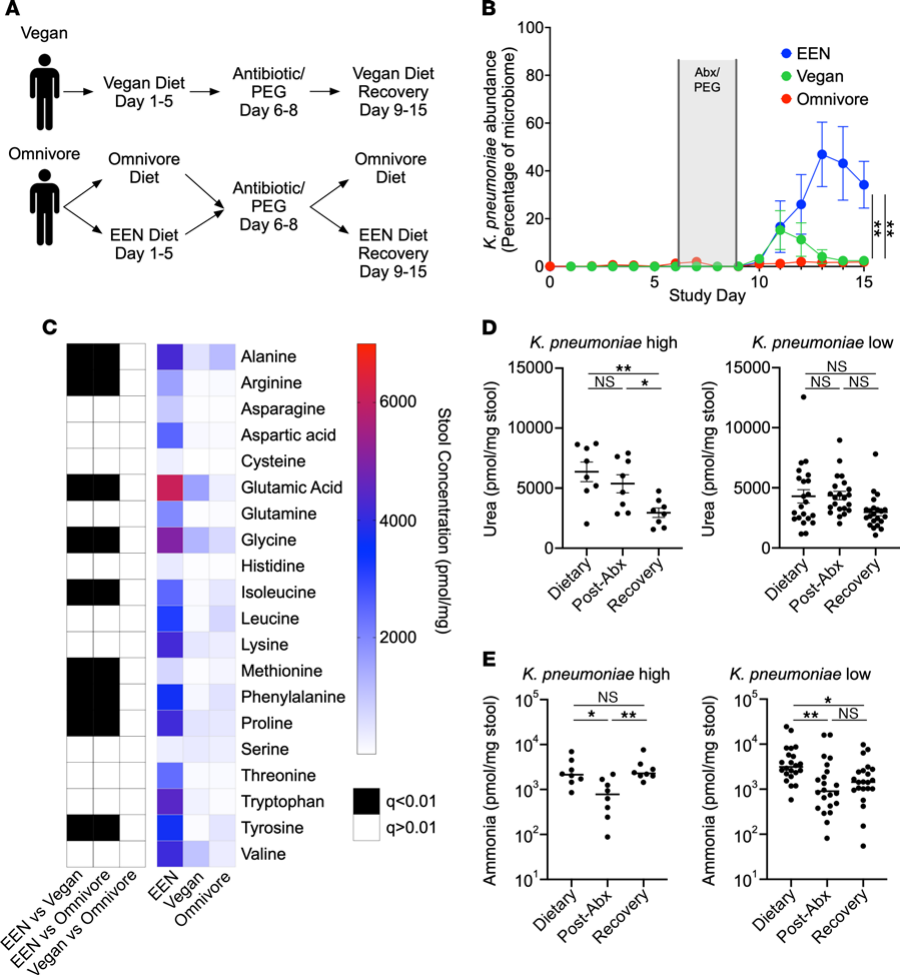
A defined formula diet favors *K*. *pneumoniae* growth in humans. (**A**) Diagram of the FARMM study design. Patients were randomized to a FF diet (EEN) or an omnivore diet; a third group remained on a vegan diet throughout the study. Recovery of the microbiome was monitored after antibiotic and PEG depletion. (**B**) Relative abundance of *K*. *pneumoniae* as a percentage of the microbiome determined via shotgun metagenomic sequencing, stratified by dietary group. (**C**) Heatmap of average stool amino acid concentrations during the microbiome recovery phase (day 14) of the FARMM study, stratified by diet. Black boxes denote a statistically significant difference of amino acid concentration between the indicated dietary groups. (**D** and **E**) Quantification of stool urea (**D**) and ammonia (**E**) at each phase of the FARMM study in samples from individuals with a high or low relative abundance of *K*. *pneumoniae* (defined as >20% *K*. *pneumoniae* by relative abundance during the recovery phase). Data are presented as the mean ± SEM. *n* = 10 participants per dietary group. *P < 0.05 and **P < 0.01, by 1-way ANOVA with Holm-Šidák’s correction for multiple comparisons (**B**), comparing the EEN versus omnivore and the EEN versus vegan groups on day 15, (**D**) multiple Mann-Whitney *U* test with the FDR method of Benjamini, Krueger, Yokutieli (**C**), or Kruskal-Wallis test with Dunn’s multiple-comparison test (**E**). Abx, antibiotics.

A previously published study from our group, referred to as the Food and Resulting Microbial Metabolites (FARMM) study, examined the effect of dietary fiber on the microbial and metabolomic composition of the gut microbiome in humans (33). Healthy individuals were recruited, of whom 20 normally adhered to a typical American diet (referred to as omnivore) and 10 to a vegan diet. The 20 omnivore participants were randomized to a standardized omnivore diet or a fiber-free (FF) exclusive enteral nutrition (EEN) diet, whereas the vegan participants remained on a vegan diet (Figure 1A). The omnivore and EEN diets were similar in macronutrient composition, except for the lack of dietary fiber in the EEN and an altered ratio of saturated and unsaturated fats. After a 5-day dietary phase, all participants were subjected to microbiota depletion via a 3-day course of nonabsorbable oral antibiotics (vancomycin and neomycin), together with a 1-day PEG purge. Subsequent recovery of the microbiome was monitored by fecal analysis including shotgun metagenomic sequencing and metabolomics. Previously published results from this study revealed that the diet lacking in fiber, EEN, reduced microbial diversity during microbiome recovery and increased the relative abundance of *Pseudomonadota* (33), reminiscent of a dysbiotic signature.

Analysis of species abundance revealed that *K*. *pneumoniae* bloomed in the EEN group, accounting for over 50% of the microbiota, which was not observed durably in either the omnivore group or the vegan group (Figure 1B and Supplemental Figure 1A; supplemental material available online with this article; https://doi.org/10.1172/JCI174726DS1). There was a brief increase in abundance noted in the vegan group, which resolved by day 13, correlating with the timing of microbiota recovery. On the final day of the trial, we noted a clear separation of participants with high levels of *K*. *pneumoniae* (*K*. *pneumoniae–*high, >20%) and those with low levels (*K*. *pneumoniae–*low, <20%) (Supplemental Figure 1A). Further analysis showed that *K*. *pneumoniae–*high individuals had a distinct microbiota composition, but no consistent correlation with other organisms was identified (Supplemental Figure 1, B and C). Prior to initiation of the study, only 1 individual had detectable *K*. *pneumoniae* via shotgun sequencing, however, it is difficult to exclude the possibility that participants were colonized at levels below the limit of detection by sequencing. These data suggest that an EEN diet provided a significant advantage to *K*. *pneumoniae* colonization during microbiota recovery.

One characteristic feature of *K*. *pneumoniae* is its production of the enzyme urease, which metabolizes host-generated urea into ammonia and has been associated with dysbiosis and inflammation in a mouse model of IBD (35). We predicted that a high abundance of *K*. *pneumoniae* would correlate with increased bacterial urease in the microbiome. Shotgun metagenomic sequencing confirmed an increase in the core urease genes in the EEN dietary group relative to the omnivore and vegan groups during microbiome recovery (Supplemental Figure 2A). Operon reconstruction found that the majority of urease was indeed attributable to *K*. *pneumoniae* in the EEN cohort (Supplemental Figure 2B). Furthermore, in the omnivore diet group during the early stage of microbiome recovery, we observed a spike of urease operon genes that were found to be from *Streptococcus thermophilus* (Supplemental Figure 2B) and likely attributable to its presence in yogurt in the omnivore diet.

### Urea favors urease-encoding organisms in a complex microbial community.

We hypothesized that *K*. *pneumoniae* colonization would alter the fecal nitrogen environment by metabolizing amino acids and urea into ammonia, thus providing a fitness advantage in colonization. During the dietary phase of the study, all 20 proteogenic amino acids were of low abundance (Supplemental Figure 2C). Antibiotic treatment increased stool amino acid levels, consistent with previously published study results (Supplemental Figure 2C) (33); however, during microbiome recovery, the EEN dietary group displayed decreased amino acid consumption relative to the omnivore and vegan groups on day 14 of the study (Figure 1C). Urea and ammonia quantification revealed that individuals with a high abundance of *K*. *pneumoniae* during microbiota recovery had decreased fecal urea and a corresponding increase in ammonia during microbiome recovery, whereas those with relatively low *K*. *pneumoniae* levels did not (Figure 1, D and E). Thus, EEN supported higher levels of *K*. *pneumoniae* engraftment in humans and altered the luminal nitrogen composition of the microbiome.

We generated an in vitro model to explore the role of urea in a complex gut microbial community. A bioreactor system was inoculated with a human fecal sample into 2 complex media: (a) brain heart infusion (BHI) medium, in which the primary carbohydrate is glucose, or (b) SHIME (Simulator of the Human Intestine Microbial Ecosystem) medium, which contains complex glycans (36). The low concentration of urea in both media provided the opportunity to directly test the effect of urea on an intestinal microbial community. Samples were collected every 24 hours and subjected to shotgun metagenomic sequencing, and relative species and gene abundances were calculated. We found that in the BHI medium, the addition of urea significantly increased the abundance of the urease-positive organisms *K*. *pneumoniae* and *Citrobacter freundii* (Supplemental Figure 3, A and B). The abundance of urease genes accordingly increased with urea supplementation (Supplemental Figure 3C). These relationships were substantially diminished in SHIME media (Supplemental Figure 3, A–C), supporting the theory that urease provides a growth advantage in the presence of urea through increased nitrogen availability only when a simple carbohydrate is available for use by *Enterobacteriaceae*.

### Nitrogen is not a limiting resource for K. pneumoniae colonization in the gut.

To rigorously test the hypothesis that nitrogen is growth limiting in the gut for *K*. *pneumoniae*, we generated 2 deletion mutants: the urease operon (Δurease) and *ntrC* (Δ*ntrC*), which encodes a key regulator of the nitrogen regulatory (Ntr) system (37). While the WT strain grew well in vitro in an ammonia-limited environment with urea as an available nitrogen source, the Δurease and Δ*ntrC* strains displayed delayed or limited growth (Supplemental Figure 3, D and E). In an ammonia-rich environment, urea had no effect on the 3 strains. When tested on a range of nitrogen-containing compounds, the Δ*ntrC* strain was significantly restricted in its growth relative to the WT strain (Supplemental Figure 4A). Thus, we found that under nitrogen-limited environments in vitro, the *K*. *pneumoniae* had a broad capacity for nitrogen assimilation that was dependent on urease and *ntrC*.

To directly test whether restricting the nitrogen resources available to *K*. *pneumoniae* reduces colonization capacity in the gut, mice were colonized with WT, Δurease, or Δ*ntrC* *K*. *pneumoniae* after antibiotic pretreatment on a standard mouse chow (Supplemental Figure 4, C and D). Interestingly, we found no colonization defect for the mutant strains, and, in fact, a previous study with *E*. *coli* observed a colonization advantage for a Δ*ntrC* mutant (38). These observations led us to speculate that under normal dietary conditions, the gut is abundant in available nitrogen. Previous work demonstrated that limitation of dietary protein significantly reduced ammonia and urea in the mouse intestine (39). However, we find that urease and *ntrC* are also dispensable for colonization of mice on a low-protein diet (Supplemental Figure 4, E and F). The absence of complex carbohydrates similarly had no effect on colonization of Δurease *K*. *pneumoniae* (Supplemental Figure 4, G and H). We confirmed that urease did in fact alter the intestinal nitrogen environment, increasing ammonia levels with corresponding urea consumption (Supplemental Figure 4, I–N).

**Figure 2:**
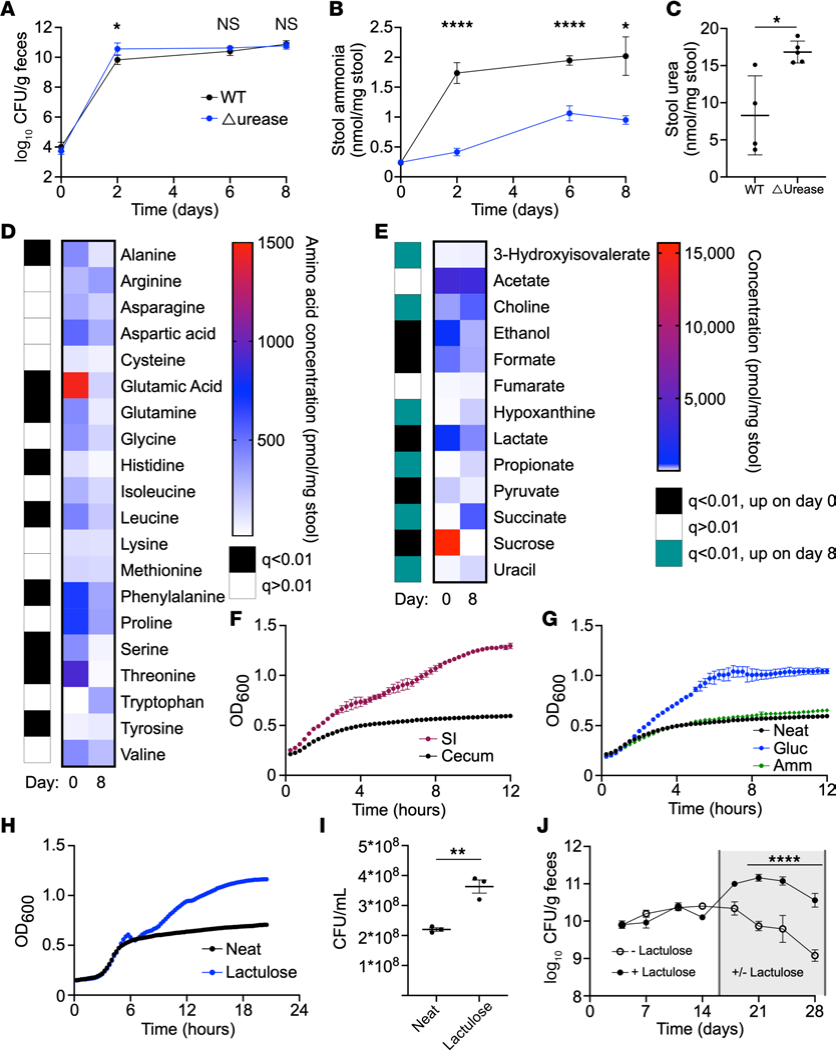
*K*. *pneumoniae* colonization is limited by carbon source availability and alters the nitrogen environment in the gut. (**A**–**E**) GF mice were colonized with WT or Δurease *K*. *pneumoniae*, and serial stool collections were done throughout the 1-week study. Fecal CFU (**A**) and stool ammonia (**B**) were monitored for 1 week after colonization. Fecal urea (**C**) was tested on day 8. Stool amino acid levels (**D**) and metabolites (**E**) were quantified from stool before (day 0) and after (day 8) WT K. *pneumoniae* colonization. (**F**) Growth of WT *K*. *pneumoniae* in small intestine (SI) or cecal extracts from mice monitored via OD_600_. (**G**–**I**) Growth in cecal extracts supplemented with ammonia (Amm) or glucose (Gluc) (**G**) or lactulose (**H** and **I**) quantified by OD_600_ and CFU (**I**). Data for neat cecal extracts are presented in both **F** and **G** for reference. (**J**) Mice colonized with *K*. *pneumoniae* were subsequently treated with lactulose in the drinking water or water-only control. Data are presented as the mean ± SEM (**A** and **J**) or the mean ± SD (**B**, **C**, and **F**–**I**). *n* = 4–5 mice per group (**A**–**E**, **J**) or *n* = 3 wells per group (**F**–**I**). Data represent combined results from 2 independent experiments (**A**–**E**) or are from a single experiment representative of 3 independent experiments (**F**–**J**). *P < 0.05, **P < 0.01, and ****P < 0.001, by multiple unpaired, 2-tailed *t* tests with Benjamini Hochberg multiple corrections (**D** and **E**), with Bonferroni’s multiple corrections (**A**, **B**, and **J**) or by unpaired, 2-tailed *t* test (**C** and **I**).

To rule out the effect of cross-feeding from other members of the microbiota on colonization of urease-null *K*. *pneumoniae*, we colonized germ-free (GF) mice with WT or Δurease *K*. *pneumoniae*. Intestinal colonization of *K*. *pneumoniae* was independent of urease expression (Figure 2A). We found that, prior to gavage, fecal ammonia levels were remarkably low (Figure 2B), similar to those of microbiome-depleted conventionally raised (CR) mice (Supplemental Figure 4J). Colonization with WT *K*. *pneumoniae* resulted in an approximately 10-fold increase in fecal ammonia levels; this was partially urease dependent as colonization with Δurease *K*. *pneumoniae* resulted in significantly less ammonia production than the WT strain but remained approximately 4-fold above the GF baseline. WT *K*. *pneumoniae* colonization caused decreased fecal urea levels relative to the urease-deficient strain, demonstrating urea hydrolysis as a primary source of ammonia production (Figure 2C). Fecal amino acid analysis revealed a significant decrease in the concentration of several amino acids after colonization with the WT strain (Figure 2D), probably due to consumption by *K*. *pneumoniae*. Combined with the production of gut ammonia by the Δurease *K*. *pneumoniae* strain, this reduction of fecal amino acids suggests the utilization of amino acids as a carbon source via deamination, thereby leading to the release of ammonia into the luminal environment.

### K. pneumoniae colonization is enhanced by increased carbon source availability.

The observed pattern of amino acid consumption and ammonia production after *K*. *pneumoniae* colonization indicated that carbon may be the primary limited resource for *K*. *pneumoniae* colonization in the gut. Through in vitro testing, we found that *K*. *pneumoniae* had a strong preference for simple carbohydrates, in addition to select amino acids, and citric acid cycle intermediates (Supplemental Figure 4B), while complex carbohydrates including inulin and dextrin are not utilized. Proton nuclear magnetic resonance (NMR) quantification of fecal samples before and after colonization of GF mice identified several metabolites that were significantly reduced in concentration after *K*. *pneumoniae* colonization, including sucrose, pyruvate, and lactate (Figure 2E), all of which serve as carbon sources for *K*. *pneumoniae* in culture (Supplemental Figure 4B). These data support the idea that *K*. *pneumoniae* is carbon limited in the intestinal environment and that alternative sources of nitrogen are unnecessary for colonization.

The mammalian small intestine absorbs most simple carbohydrates and amino acids, supporting the hypothesis that *K*. *pneumoniae* is limited by carbon sources available in the colon. We generated an ex vivo model in which the small intestinal or cecal contents of microbiota-depleted CR mice were extracted, sterilized, and used as culture media for growth of *K*. *pneumoniae*. Growth in the small intestinal extract was greater than that in the cecal content, suggesting that host-absorbed nutrients were important for intestinal growth of *K*. *pneumoniae*. Addition of the readily usable nitrogen source, ammonia, to cecal extract had no effect on growth of *K*. *pneumoniae*, however, glucose supplementation increased the maximum density and growth rate (Figure 2, F and G) to mirror that of the small intestinal extract.

One simple carbohydrate that is not absorbed or metabolized by the host is lactulose, an artificial disaccharide of galactose and fructose. We found that lactulose could be utilized as a sole source of carbon in minimal media for the tested strain of *K*. *pneumoniae* (Supplemental Figure 4B). We confirmed that addition of lactulose to the ex vivo cecal extract increased the growth of *K*. *pneumoniae*, as measured by OD and CFU (Figure 2, H and I). Mice were provided lactulose in the drinking water after colonization with WT *K*. *pneumoniae*. Compared with water control, lactulose increased *K*. *pneumoniae* colonization by 10-fold (Figure 2J). In total, these data provide strong evidence that colonization of *K*. *pneumoniae* in the gut microbiome was carbon restricted and that nitrogen was abundant in the intestinal environment.

### Dietary fiber suppresses colonization of K. pneumoniae and supports commensal competitors.

**Figure 3:**
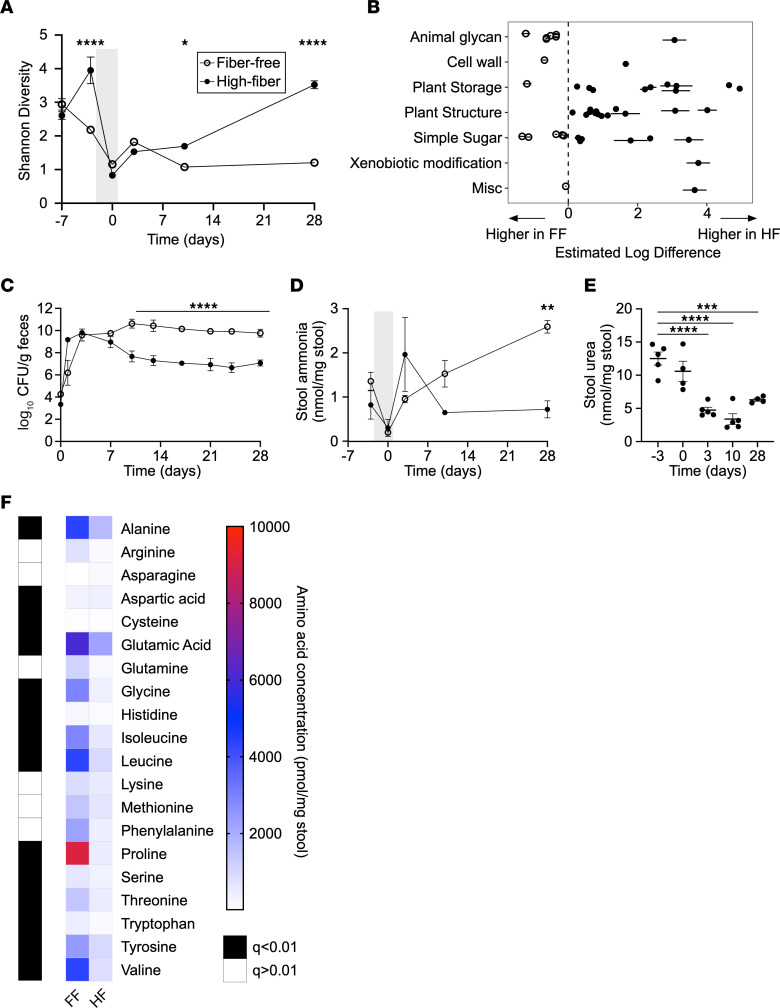
Complex carbohydrates increase microbiome diversity and reduce *K*. *pneumoniae* colonization after antibiotic depletion. (**A**–**F**) Mice were provided a FF or a HF diet starting on day –7, treated with oral antibiotics on day –3 to day 0 (gray shading), and gavaged with *K*. *pneumoniae* (day 0). Serial stool samples were subjected to analysis as follows: (**A**) Shannon α diversity of stool microbiome from mice provided a FF or HF diet including the antibiotic treatment period (gray shading) and recovery, as determined by shotgun metagenomic sequencing. (**B**) GH genes with significantly different abundances between FF and FH diets 4 weeks after gavage with *K. pneumoniae* grouped by substrate type. Open circles represent genes with higher levels in mice on the FF diet, and closed circles represent genes with higher levels in mice on the HF diet. (**C**) *K*. *pneumoniae* fecal CFU for mice on a FF (open circles) or HF (closed circles) diet measured 4 weeks after *K*. *pneumoniae* gavage. (**D**) Stool ammonia was quantified before and after antibiotic treatment (gray shading) and *K*. *pneumoniae* gavage for mice subjected to a FF (open circles) or HF (closed circles) diet. (**E**) Stool urea levels were quantified for mice provided a FF diet. (**F**) Heatmap of amino acid concentrations in mice on a FF or HF diet after colonization with *K*. *pneumoniae*. Data are presented as the mean ± SD (**A**–**E**). *n* = 5 mice per group. Data are representative of 2–3 independent experiments (**C**–**F**). *P < 0.05, **P < 0.01, ***P < 0.001, and ****P < 0.0001, by multiple 2-tailed *t* tests with Bonferroni’s correction for multiple comparisons (**A**, **C**, and **D**), 1-way ANOVA with Bonferroni’s correction for multiple comparisons (**E**), or multiple Mann-Whitney *U* tests with the FDR method of Benjamini, Krueger, and Yokutieli (**F**).

While available simple carbohydrates support the growth of *K*. *pneumoniae* in the gut, the majority of carbohydrates in the colon are complex and not absorbed by the host. We hypothesized that these complex carbohydrates, referred to as dietary fiber, may play a role in colonization resistance against *K*. *pneumoniae*. Indeed, results of the FARMM study demonstrate that the EEN diet, which is deficient in fiber, increased colonization of *K*. *pneumoniae* (Figure 1B). To test this hypothesis, we generated matched FF and high-fiber (HF) mouse chows; the HF chow contained a well-characterized pea fiber (40). Groups of CR mice were provided FF or HF chow ad libitum, treated with the same oral antibiotic regimen provided to the FARMM participants for 3 days to deplete the microbiota, and gavaged with *K*. *pneumoniae*. Calorie intake was equivalent between the groups, without significant differences in macronutrient consumption (Supplemental Figure 5A). As expected, we found that antibiotic treatment significantly reduced microbiome diversity when compared using shotgun sequencing (Figure 3A). During recovery, microbiome diversity was dependent on dietary fiber, whereby the mice provided a HF diet returned to baseline diversity by 4 weeks, whereas those on a FF diet did not. These data mirror those of the FARMM study, in which the study participants on a FF EEN diet did not recover to baseline diversity after microbiome depletion. Principal coordinate analysis (PCoA) confirmed that, although antibiotic treatment significantly affected the microbiota community structure regardless of diet, the community in mice on the FF diet remained persistently altered from baseline and from that of the HF diet group (Supplemental Figure 5B). We hypothesized that fiber would enable asymmetric recovery of organisms encoding GHs necessary for complex glycan metabolism. Indeed, while mice on a HF diet had increased levels of GHs enabling metabolism of plant-based carbohydrates, these levels were decreased in mice fed a FF diet (Figure 3B, Supplemental Figure 6A). In contrast, GHs for simple carbohydrates and animal glycans were disproportionately increased in the FF diet group, reminiscent of results from the FARMM study and consistent with the ability of a FF diet to select for a mucolytic community (41).

After gavage with *K*. *pneumoniae*, we found a 1,000-fold higher level of colonization in the FF group compared with the HF group (Figure 3C). Intriguingly, this difference only manifested after 1 week, corresponding with a divergence in microbiome diversity between groups. Species analysis of the microbiome through shotgun metagenomics identified several organisms that were permanently lost on the FF diet, the recovery of which on a HF diet anticorrelated with *K*. *pneumoniae* (Supplemental Figure 5, C and D). Several members of the microbiota including *Lactobacillus johnsonii*, *Bifidobacterium pseudolongum*, and a *Lachnoclostridium* species demonstrated a strong correlation with resistance to *K*. *pneumoniae* colonization (Supplemental Figure 5, C and D), suggesting that these species may play a causative role in the effect of this dietary intervention. Analysis of metagenomic data from the FARMM trial revealed a similar pattern, in which *Lactobacillus* and *Bifidobacterium* species were at lower levels in the EEN dietary group compared with the fiber-containing diet groups (Supplemental Figure 6C). Conversely, the pathobiont *Enterococcus faecalis* was detected at high levels in mice on the FF diet 4 weeks after antibiotic treatment, suggesting that these commensal strains may provide colonization resistance more broadly in the setting of dietary fiber.

Examination of the nitrogen composition of the gut demonstrated that stool ammonia was increased in the FF group, corresponding to higher levels of *K*. *pneumoniae,* and was associated with urea depletion over the course of the study (Figure 3, D and E). By contrast, we did not find such a relationship in the HF diet group (Figure 3D and Supplemental Figure 6B), similar to the results of the FARMM study (Figure 1, D and E). Moreover, we observed higher levels of stool amino acids in the FF group relative to the HF group during microbiome recovery (Figure 3F), suggesting that organisms abundant in mice on the HF diet preferentially consumed amino acids. In summary, through mouse modeling in which dietary fiber was removed, we have replicated several key features of the FARMM study conducted in humans, including altered microbiome diversity, GH abundance, *K*. *pneumoniae* colonization, and nitrogen composition. Our results demonstrate that complex dietary carbohydrates played a key role in colonization resistance against *K*. *pneumoniae*.

### Dietary fiber reduces bacterial dissemination after intestinal barrier disruption.

**Figure 4:**
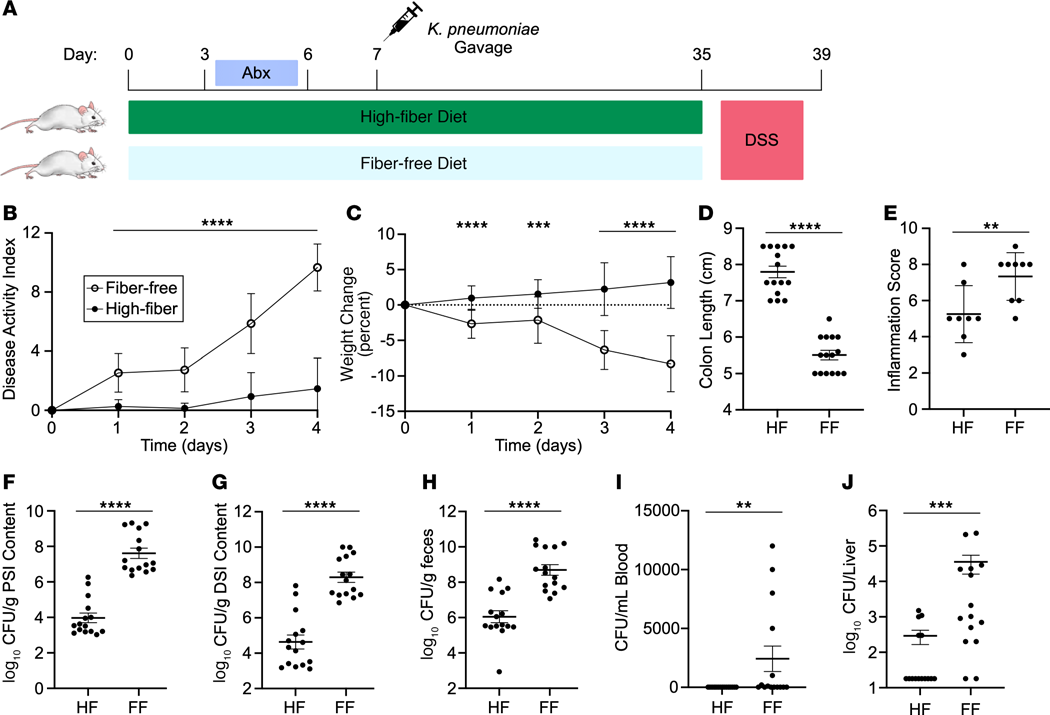
A HF diet protects against DSS colitis and *K*. *pneumoniae* dissemination from microbiota. (**A**) Diagram of experimental design. Mice were placed on a HF or a FF diet and colonized with WT *K*. *pneumoniae* after antibiotic pretreatment followed by treatment with 5% DSS in the drinking water. (**B** and **C**) Disease activity (**B**) and weight change (**C**) were monitored during DSS treatment. After 4 days of treatment, mice were euthanized. (**D**) Colon length was quantified. (**E**) Colon tissue was microscopically scored for evidence of inflammation. (**F**–**J**) CFU were quantified in the proximal small intestine (**F**), distal small intestine (**G**), feces (**H**), blood (**I**), and liver (**J**). Data are presented as the mean ± SD. Results are a combination of 3 independent experiments (**B**, **C**, and **F**–**J**; *n* = 15 mice per group) or a combination of 2 independent experiments (**E**; *n* = 8–9 mice per group). **P < 0.01, ***P < 0.001, and ****P < 0.0001, by multiple unpaired, 2-tailed *t* tests with Bonferroni’s multiple correction (**B** and **C**), unpaired, 2-tailed *t* test (**D**–**H**), or Mann-Whitney *U* test (**I** and **J**).

To test the effect of dietary fiber on bacterial translocation, mice were colonized with *K*. *pneumoniae* on a FF or HF diet after antibiotic pretreatment. After stable engraftment, mice were treated with dextran sodium sulfate (DSS) to induce intestinal inflammation and disrupt the epithelial barrier (Figure 4A). Consistent with previous studies, we found that disease activity, weight loss, and colon length were improved by the addition of dietary fiber (Figure 4, B–D) (42, 43). Colon histopathology revealed a concordant reduction in the inflammation score in the HF diet group (Figure 4E). After 4 days of treatment, mice were euthanized, and *K*. *pneumoniae* were quantified from sections of the intestinal tract, liver, and blood. Dietary fiber not only reduced colonization of *K*. *pneumoniae* in the colon but also in the proximal and distal small intestine (Figure 4, F–H). We observed that fiber prevented the dissemination of *K*. *pneumoniae* to the liver and blood and that mice treated with a FF diet had higher levels of bacteremia and liver infection (Figure 4, I and J). In total, our data unveil a role for dietary carbohydrates in the colonization of *K*. *pneumoniae* in the gut and provide a possible treatment to reduce dissemination of this human pathogen.

## Discussion

Dysbiosis is an important feature of many chronic diseases and involves the outgrowth of *Enterobacteriaceae* in the gut. Intestinal colonization of *K*. *pneumoniae* and other *Enterobacteriaceae* is thought to play a role in IBD and disseminated infectious diseases including bacteremia, cholangitis, and urinary tract infections. Identification of the dietary components contributing to the formation and resolution of dysbiosis is therefore critical. Here, we found that diet could have a direct effect on colonization of *K*. *pneumoniae* by serving as a nutrient source and an indirect effect via colonization resistance. Understanding the dietary resources necessary for colonization of harmful strains of *Enterobacteriaceae* could serve as a critical tool in targeted prebiotic interventions to prevent and/or treat disease.

Since both nitrogen and carbon sources are critical for bacterial growth, and the environment of the distal gut is known to be a nutrient constrained environment (28), we systematically determined the relative importance of nitrogen- versus carbon-based nutrients in the colonization of *Enterobacteriaceae* in both the murine and human gastrointestinal (GI) tract. Previous literature suggests that nitrogen is a limiting resource for bacterial growth in the mammalian gut (29, 30). Urea is a host-produced nitrogenous waste product that is in turn excreted into the intestinal lumen and has long been speculated to be an important source of nitrogen for the microbiome, accessed via bacterial urease (28, 44). Recent literature shows that bacterial urease is important for cross-feeding of nitrogen to non-urease-encoding species (31). Our human and bioreactor data suggested that urease may be advantageous for the growth of *K*. *pneumoniae* in microbiome recovery, specifically when the readily accessible nitrogen source, ammonia, is less abundant. However, after rigorous testing in vitro, ex vivo, and in vivo, we found that nitrogen was not a limiting resource for the colonization of *K*. *pneumoniae*. Even under nitrogen-starved conditions, bacterial urease and the nitrogen scavenging system Ntr are dispensable for growth of *K*. *pneumoniae*. Intriguingly, the presence of oligosaccharides correlates with a diminished effect of urea on the microbial composition in complex media. Determining if these carbohydrates mediate the nitrogen effect observed will require further studies, given the many differences between these complex media with limited characterization. Overall, our data suggest that nitrogen is abundant in the mouse intestinal tract and that other dietary factors are more central for the colonization of this human pathogen.

Regardless, *K*. *pneumoniae* colonization has a significant effect on the nitrogen environment of the intestine. Only select amino acids are consumed after *K*. *pneumoniae* mono-association of GF mice, including serine and threonine, which are most likely being metabolized as carbon sources through deamination. *K*. *pneumoniae–*colonized GF mice maintain higher levels of many amino acids compared with CR mice or humans with intact microbiota; other members of the community, particularly *Clostridia*, are capable of Stickland fermentation and thus have a broader accessibility to energy extraction from amino acids (45). Interestingly, we found that luminal ammonia, which serves as a preferred nitrogen source for most bacterial species, was dependent on the presence of the gut microbiome, whereas feces of GF mice and antibiotic-treated mice and humans contained substantially lower levels. Our work demonstrates that, although urease was a factor in ammonia production during colonization in GF mice, ammonia was also released from other sources, likely including amino acid deamination (46). While our conclusion regarding nitrogen abundance is limited to *K*. *pneumoniae*, it is plausible that other bacterial species in the gut microbiome are nitrogen limited (28).

In contrast to our findings with nitrogen availability, our studies show that carbon sources were a major limiting factor for the growth of *K*. *pneumoniae* in the gut. The host absorbs most simple carbohydrates in the small intestine, leaving a relative paucity of dietary simple carbohydrates available to the microbiota in the colon (47–49). Previous work focused on *E*. *coli* has identified multiple carbohydrate utilization genes important for competitive colonization of the GI tract, including gluconate, mannose, fucose, and ribose (50), which are thought to be liberated from host mucus polysaccharides (51). While *K*. *pneumoniae* carbohydrate consumption in the intestine is less well studied, recent work demonstrated that fucose utilization is important for intestinal colonization (52). The role of dietary carbohydrates in the colonization of *Enterobacteriaceae* has not been well characterized to date. Our ex vivo modeling revealed that growth in the small intestinal luminal extract was greater than that in the cecal extract, the effect of which was diminished with the addition of a simple carbohydrate to the cecal material. Host absorption of simple carbohydrates in the small intestine may limit their effect on the colonization of *Enterobacteriaceae* in the colon. However, supplementation of the diet with a nonabsorbed carbohydrate, lactulose, in a mouse model increased the colonization of *K*. *pneumoniae*, consistent with the hypothesis that carbohydrates are a major limiting resource for colonization.

In contrast to simple carbohydrates, we found that complex carbohydrates in dietary fiber suppressed the colonization of *K*. *pneumoniae* in both humans and in mice, correlating with increased microbiome diversity. Intriguingly, we observed that the vegan and omnivore groups in the FARMM trial lacked a sustained difference in *K*. *pneumoniae* colonization, despite evidence that the vegan participants had higher dietary fiber intake (33). Thus, a dose-dependent response to dietary fiber was not clearly seen for *K*. *pneumoniae* colonization resistance. Two non-mutually exclusive hypotheses may explain this phenotype. First, it is plausible that dietary fiber reached a threshold, whereby above a particular quantity, the effect of increased fiber diminished. Indeed, previous human studies support this concept (53–55). Second, the effect of fiber was personalized and dependent on the fiber type and baseline microbiota, which also has support from a previous clinical trial (56). Future work will be necessary to clarify the inter-subject variability observed in response to fiber.

Consistent with the FARMM study, dietary fiber was associated with an enrichment for plant-based GH abundance in the fecal microbiome, whereas a FF diet favored GHs for simple carbohydrates and animal glycans (33). Short-chain fatty acids (SCFAs), often produced in the intestine from dietary fiber, have been shown to suppress *K*. *pneumoniae* growth via intracellular acidification (57). This suggests that dietary fiber may have 2 roles in inhibiting *Enterobacteriaceae* colonization: directly through SCFA production and by supporting the recovery of competing organisms encoding plant saccharide GHs. *Clostridioides difficile* colonization is somewhat analogous, as it also relies on both depletion of microbiome diversity and decreased SCFA production (58). Our results show that fiber not only affected colonization of *K*. *pneumoniae* in the colon but also in the proximal and distal small intestine during DSS colitis. As a member of the *Enterobacteriaceae* family, the relatively bile- and oxygen-rich environment of the small intestine is likely to favor *K*. *pneumoniae* growth (59). This finding could have been influenced by coprophagia, however, which has been shown to significantly alter small intestinal microbiota composition in mice (60). Further studies will be necessary to define the causal strain(s), the essential GHs, and the role of coprophagia in this phenotype.

The suppression of *K*. *pneumoniae* colonization in the microbiome may have significant disease implications. For patients with IBD, a reduction of *K*. *pneumoniae* colonization improved the host inflammatory response (20). Moreover, disseminated *K*. *pneumoniae* infections, in part, derive from the gut microbiome (61). Indeed, we found that disruption of the intestinal barrier with DSS led to the dissemination of *K*. *pneumoniae* to the liver and bloodstream, which was nearly eliminated by a reduction of colonization with dietary fiber. Lack of fiber in the diet has separately been shown to increase the severity of DSS and infectious colitis, and thus the effect observed was likely multifactorial (41, 42). The effect of fiber on IBD has been the subject of significant controversy, with evidence demonstrating both benefits and risks associated with complex carbohydrate supplementation for these patients. Several recent studies in humans and mice suggest that the effect of fiber on inflammation is carbohydrate, microbiota, and model dependent (56, 62, 63). Although our data show a role for dietary fiber in the suppression of acute colitis, future work will be required to demonstrate if this is applicable to chronic inflammation.

Our studies reveal the critical importance of dietary carbohydrates in the colonization of *K*. *pneumoniae* in the mammalian gut. This work may have direct relevance to human disease; patients hospitalized in the intensive care unit often require EEN as their sole source of nutrition, which is typically given without fiber supplementation. The outgrowth of *Enterobacteriaceae* in this setting due to poor microbiome diversity may predispose these patients to disseminated infection. Resolution of dysbiosis through rational prebiotic and probiotic therapies would have a range of therapeutic applications. Understanding the limiting metabolic resource(s) for these disease-associated organisms and the effect of diet on their colonization is a key step in designing such treatment strategies.

## Methods

### Sex as a biological variable.

Our human study examined both male and female participants, and the combined findings are presented in this work where there was no significant difference in the results between males and females. The mouse modeling was conducted in female mice only to reduce variability and because results were expected to be relevant regardless of biological sex based on the results of our human study.

### Bacterial strains, culture conditions, and antibiotics.

The *K*. *pneumoniae* strain used was MGH 78578, as noted in Supplemental Table 1. *K*. *pneumoniae* was grown in LB (Miller) medium aerobically at 37°C unless otherwise noted. M9 minimal media were used with carbohydrate or nitrogen supplementation as follows: 0.1% ammonia, 0.2% glucose, or 0.2% lactulose where noted. The following antibiotics were used: kanamycin, apramycin, vancomycin, and hygromycin at concentrations of 25, 100, 8, and 50 μg/mL, respectively.

### Strain construction.

Deletion of the indicated genes in the MGH 78578 *K*. *pneumoniae* strain was performed using recombineering through replacement of the targeted genes with a FRT-apramycin-FRT construct and subsequent excision, as previously described (64). In brief, the pMDIAI plasmid served as the template for PCR amplification of the apramycin resistance cassette, flanked by FRT sites and a 50 bp overlap region with the target gene. Electrocompetent *K*. *pneumoniae* was transformed with pACBSR-hyg for recombineering (65). The PCR-amplified cassette was purified and electroporated into this strain with 1 μg DNA and plated on LB-apramycin. The pFLP-hygromycin plasmid was used to excise the antibiotic resistance cassette, and all plasmids were cured from the strain through serial growth. PCR confirmed the mutant strains. See Supplemental Tables 1 and 2 for plasmid information and oligonucleotide sequences.

### Analysis of nitrogen and carbon sources.

To determine the sole carbon and nitrogen sources available to *K*. *pneumoniae*, the WT or Δ*ntrC* strains were grown overnight in M9 minimal media with ammonia and glucose, washed in PBS, diluted 1:1,000 into M9 minimal media without ammonia (nitrogen testing) or without glucose (carbon testing). Overnight-grown culture was diluted 1:1,000 in sterile PBS and aliquoted into Biolog Phenotype microarray 96-well plates PM1, PM2A (carbon testing), and PM3B (nitrogen testing). OD_600_ was determined via the BioTek Epoch2 plate reader and grown for 24 hours with shaking at 37°C. The AUC was calculated using GraphPad Prism 9.5.1 (GraphPad Software) after subtracting background absorbance.

### Bioreactor model.

Eppendorf Bioflo 320 bioreactors were assembled according to the manufacturer’s specifications. During the experiment, all bioreactors were maintained at a temperature of 37°C with agitation set to 100 rpm. The pH was kept at 7 ± 0.1, using 1 M NaOH and CO_2_ gas. There was a constant sparging of gas at a rate of 1 L/min, with the ratio of N_2_/CO_2_ dependent on the pH control. The initial volume of the bioreactors was 750 mL of either BHI broth or a 70:30 ratio of adult M-SHIME growth medium/pancreatic juice. Adult M-SHIME growth medium with starch was purchased from ProDigest (Ghent) and consists of arabinogalactan (1.2 g/L), pectin (2 g/L), xylan (0.5 g/L), glucose (0.4 g/L), mucin (2 g/L), and starch (4 g/L), as well as yeast extract (3 g/L) and peptone (1 g/L), which provide nitrogen and trace nutrients (66). Pancreatic juice was made fresh every 2–3 days and contained 12.5 g NaHCO_3_ (MilliporeSigma), 6 g oxgall bile (BD), and 0.9 g pancreatin (MilliporeSigma) (36). The bioreactors were connected by silicon tubing (Cole Parmer) to a supply of medium and pancreatic juice to provide nutrition and biliary pancreatic enzymes and to a bard urinary drainage bag (BD) to collect waste. For inoculation, fecal samples were suspended at 10% wt/v in phosphate buffer within an anaerobic chamber. Buffer consisted of 0.8 g K_2_HPO_4_, 6.8 g KH_2_PO_4_, 0.1 g sodium thioglycolate, and 15 mg sodium thionite per liter, with pH adjusted to 7. At the time of inoculation, 10 mL fluid was removed from the bioreactor, and 10 mL of the resuspended inoculums was added. The bioreactors were grown overnight for 16 hours, with temperature, pH, agitation, and gas flow. Following overnight growth, the bioreactors were maintained with daily feeding cycles, in which the bioreactor was provided fresh medium and pancreatic juice. Every 8 hours, the volume of the bioreactor was reduced to 600 mL and 150 mL of either fresh BHI or a 70:30 adult M-SHIME growth medium/pancreatic juice mixture was added. The residence duration was 40 hours.

Reactors were inoculated with a baseline fecal sample from a healthy individual enrolled in a previously described dietary intervention study (33). Following a 14-day period at baseline conditions, urea was supplemented in the feed at a concentration of 10 mM for an additional 14-day period. Samples were collected in the morning, prior to the beginning of the feeding cycle.

### Study participants.

Stocked human fecal samples from the FARMM study were tested where noted (33). In brief, the FARMM study consisted of 30 healthy individuals, 10 of whom were vegan at baseline and 20 of whom had a typical Western (omnivore) diet. Vegan participants maintained an outpatient vegan diet during the study. Participants in the omnivore group were randomized to a standardized omnivore diet or the EEN diet consisting of Modulen IBD (Nestle). The omnivore standardized diet was designed to have a similar composition, except for the lack of dietary fiber in the EEN diet. All participants underwent 3 days of oral vancomycin 500 mg and neomycin 1,000 mg every 6 hours on days 6, 7, and 8 of the study. All participants were also given a bowel purge consisting of 4 L PEG purgative (GoLytely).

### Mice.

SPF C57Bl/6J female mice were purchased from The Jackson Laboratory at 8 weeks of age. Mice were housed under standard lighting cycle conditions (12 hours on/12 hours off) and provided acidified water. There was no investigator blinding for these studies, and no animals were excluded from analysis.

### Mouse K. pneumoniae colonization model.

All CR mice were specific pathogen–free (SPF) C57Bl/6J strain females acquired from The Jackson Laboratory at 8 weeks of age. For colonization with *K*. *pneumoniae*, mice were treated with the antibiotics vancomycin (2.5 g/L) and neomycin (5 g/L) with aspartame (25 g/L) in the drinking water provided ad libitum for 72 hours. Mice were then orogastrically gavaged once with 10^8^ CFU *K*. *pneumoniae* in 100 μL, the inoculum of which was generated from aerobically grown overnight culture in LB media and then washed and diluted 1:10 in sterile PBS. Fecal samples for CFU analysis were collected, homogenized in PBS, serially diluted, and spot plated on LB agar supplemented with kanamycin and vancomycin or with MacConkey agar supplemented with kanamycin, vancomycin, and tetracycline. Plates were incubated for 12 hours at 37°C, colonies were counted, and CFU were calculated per weight of fecal sample. C57Bl6/J mice from The Jackson Laboratory have been previously shown to not be colonized with *Enterobacteriaceae* at baseline, which limited background signal via this method (67). Prior to and after antibiotic treatment, but before gavage, each mouse was routinely tested for resistant colonies.

Fecal samples collected for metabolomics analysis were flash-frozen in dry ice and stored at –80°C. Diets were irradiated and provided ad libitum. The following diets were used in the indicated experiments: standard diet (Research Diets, AIN-76A), FF diet (TestDiet 5Z6G), HF diet (TestDiet 5Z6L, containing Vitacel Pea Fiber EF-100 from J. Rettenmaier), and a low-protein diet (Research Diets, D08092201).

For lactulose treatment experiments, mice were colonized with *K*. *pneumoniae* and subsequently provided 5% lactulose w/v in the drinking water ad libitum after 2 weeks of colonization.

### Mouse DSS and dissemination model.

For the DSS colitis model, mice were treated with 5% DSS in the drinking water. Mice were monitored for disease activity, similarly to previously published protocols (68). In brief, mice were scored daily on the criteria outlined in Supplemental Table 3. The scores of each subsection were summed to provide a total daily score for each mouse, with a maximum daily score of 12. After 4 days of treatment, mice were euthanized, and liver and blood were harvested. Luminal content was collected at the time of euthanasia from the proximal 10 cm of small intestine, the distal 10 cm of small intestine, and fecal pellets. Colon length was measured at the time of euthanasia. Liver samples and intestinal luminal contents were homogenized, and all samples were serially diluted and spot plated for CFU on LB with kanamycin and vancomycin. CFU were calculated per weight or per liver, as indicated.

For evaluation of colonic microscopic inflammation after DSS treatment, colonic tissue was dissected, opened on the antimesenteric margin, and Swiss rolled. The tissue underwent fixation in 10% formalin for 24 hours and was washed and stored in 70% ethanol. Tissue was paraffin embedded, sectioned, and stained with H&E. Tissue sections were evaluated for inflammation by an independent pathologist in a blinded fashion, using a modified scoring system from previous literature (Supplemental Table 4) (69, 70).

### GF mouse colonization model.

For GF animal experiments, female C57Bl/6J mice were raised and maintained in sterile conditions in the University of Pennsylvania Gnotobiotic Facility. For colonization, mice were orogastrically gavaged at 8 weeks of age with the indicated *K*. *pneumoniae* strains. Fecal samples were harvested before and after gavage at the indicated time points and subjected to noted CFU and metabolomics analysis.

### Ex vivo model.

Eight-week-old female C57Bl/6J mice were obtained from The Jackson Laboratory and treated for 72 hours with antibiotics as previously noted and in the final 24 hours with 10% PEG in the drinking water. Regular water was then resumed for 24 hours. Next, mice were euthanized, and small intestinal or cecal luminal content was harvested, weighed, and resuspended in M9 minimal media without ammonia or glucose supplementation at a ratio of 4 mL:1 g material. Samples were homogenized for 10 minutes and centrifuged at 10,000*g* for 10 minutes twice. Supernatant was filter sterilized through a syringe-driven 0.45 μm PTFE filter. For aerobic experiments, an overnight aerobic culture of *K*. *pneumoniae* grown in LB was washed in PBS, diluted 1:1,000, and inoculated into 200 μL small intestine or cecal extract in 96-well plates. For anaerobic experiments, small intestine or cecal extracts were allowed to equilibrate in an anaerobic chamber for 24 hours and inoculated with a PBS-washed anaerobic overnight culture of *K*. *pneumoniae*, diluted 1:1,000. Growth was monitored for 24 hours via OD_600_ in a BioTek Epoch2 plate reader, incubated at 37°C. CFU counts were obtained via serial dilution and spot plating on LB agar with kanamycin and vancomycin after 24 hours of growth.

### Shotgun sequencing and analysis.

DNA was extracted from stool using the QIAGEN DNeasy PowerSoil Pro kit for the mouse experiments and QIAGEN DNeasy PowerSoil for the cultivar experiments. Extracted DNA was quantified using the Quant-iTTM PicoGreen dsDNA assay kit (Thermo Fisher Scientific). Shotgun libraries were generated from 7.5 ng DNA using IDT for Illumina unique dual indexes and Illumina DNA Prep Library Prep kit for the mouse experiments and Illumina XT for the cultivar experiments at a 1:4 scale reaction volume. Library success was assessed by a Quant-iT PicoGreen dsDNA assay. Equal volumes of library were pooled from every sample and sequenced using a 300-cycle Nano Kit on the Illumina MiSeq. Libraries were then repooled on the basis of demultiplexing statistics of the MiSeq Nano run. Final libraries were quality controlled on the Agilent BioAnalyzer to check the size distribution and absence of additional adapter fragments. Libraries were sequenced on an Illumina NovaSeq 6000 version 1.5 flow cell for the mouse experiments and an Illumina HiSeq 2500 version 4 flow cell for the cultivar experiments, producing 2 × 150 bp paired-end reads. Extraction blanks and nucleic acid–free water were processed along with experimental samples for empirical assessment of environmental and reagent contamination. A laboratory-generated mock community, consisting of DNA from *Vibrio campbellii* and Lambda phage, was included as a positive sequencing control.

Shotgun metagenomics data were analyzed using Sunbeam, version 2.1.1 (71). Quality control steps were performed by the default workflows in Sunbeam, which included removing adapters, reads of low sequence complexity, and host-derived sequences. The abundance of bacteria was estimated using Kraken (72). Reads were mapped to the KEGG database to estimate the abundance of bacterial gene orthologs (73). Contigs were assembled from the samples in the FARMM data set using MegaHit, version 1.1.3 (74). The genes were predicted from the contigs using Prodigal, version 2.6.3 (75). The predicted gene sequences were aligned against the KEGG database using diamond aligner (76).

Within sample diversity was assessed using the Shannon diversity metric. Between sample similarity was assessed by Bray-Curtis distance. Community-level differences between sample groups were assessed using the permutational multivariate analysis of variance (PERMANOVA) test. Differences in bacterial abundance or gene orthologs were assessed using linear models on log_10_-transformed relative abundances or reads per kilobase per million mapped reads (RPKM) values. Only bacteria with 1% mean relative abundance in at least 1 comparison were tested. *P* values from multiple testing procedures were corrected to control for a specified FDR using the Benjamini-Hochberg method. A cutoff of 20% of the total microbiota during the recovery phase, based on shotgun sequencing, was used for analysis of high and low *K*. *pneumoniae* subcategorization. Once subcategorized, ammonia and urea levels were compared during the 3 dietary phases for each subgroup.

### Metabolite quantification.

Amino acids were quantified as previously described using a Waters Acquity uPLC System with an AccQ-Tag Ultra C18 1.7 μm 2.1 × 100 mm column and a Photodiode Detector Array (35). Fecal samples were homogenized in methanol (5 μL/mg stool) and centrifuged twice at 13,000*g* for 5 minutes. Amino acids in the supernatant were derivatized using the Waters AccQ-Tag Ultra Amino Acid Derivatization Kit (Waters Corporation) and analyzed using the UPLC AAA H-Class Application Kit (Waters Corporation) according to the manufacturer’s instructions. All chemicals and reagents used were mass spectrometry grade.

Stool urea quantification was performed with the Quantichrom urea kit as follows: stool samples were homogenized in 10 μL ddH_2_O per milligram stool. Solid debris was removed via centrifugation at 2,500*g* for 10 minutes. The supernatant was tested for urea quantity per the manufacturer’s protocol.

Stool metabolites were quantified using ^1^H NMR–based metabolomics as follows: approximately 50 mg wet stool sample was weighed and extracted using phosphate-buffered solution containing 50% D_2_O with 0.29 mM trimethylsilylpropanoic acid (TMSP) as an internal standard. The ^1^H spectra of extracts were acquired at 298 K using a Bruker Avance NEO 600 MHz spectrometer equipped with a SampleJet sample changer (Bruker Biospin). The noesygppr1d pulse sequence was used for recording ^1^H 1D NMR experiments with presaturation water suppression during relaxation and mixing time. All ^1^H NMR spectra were processed automatically with Chenomx NMR Suite (Chenomx Inc., version 9.05), and then each spectrum was checked and adjusted manually for phase and baseline. Metabolites were identified and spectral fit using an in-house metabolite library. Metabolite concentrations were calculated according to an internal standard for further statistical analysis.

### Statistics.

GraphPad Prism (version 10.2.1) was used to perform statistical analysis. One-way ANOVA was used for comparison of *K. pneumoniae*, *Lactobacillus*, and *Bifidobacterium* abundances between dietary groups in the FARMM study and stool urea levels across multiple time points. Multiple unpaired, 2-tailed *t* tests were used for comparison of stool amino acid and metabolite levels. The Kruskal-Wallis test was used for comparison of stool ammonia levels. Multiple 2-tailed *t* tests were used for comparison of fecal CFU, disease activity, mouse weights, mouse dietary macronutrient consumption, amino acids, and ammonia levels across multiple time points. Two-tailed *t* tests were used to compare CFU between groups from intestinal contents, liver, and blood tissues. A linear model was performed on log_10_-transformed abundances from bioreactor sequencing data. A *P* value of less than 0.05 was considered statistically significant. Data are presented as the mean ± SD or the mean ± SEM, as noted in the figure legends.

### Study approval.

All animal studies were conducted in accordance with ethics regulations under protocols approved by the University of Pennsylvania IACUC and Biosafety Committees. The University of Pennsylvania IRB approved the FARMM study protocol and considered it exempt from the clinical trial registration requirement. Written informed consent was received from participants prior to their participation in the study.

### Data availability.

Shotgun sequencing data from the FARMM study are publicly available as previously reported in the Sequence Read Archive (SRA) (PRJNA675301).

Shotgun sequencing data from this study are publicly available in the SRA (PRJNA976029 and PRJNA1065816).

Data values presented in the figures are provided in the Supporting Data Values file.

### Resource availability.

Requests for resources should be directed to Gary D. Wu (gdwu@pennmedicine.upenn.edu).

### Materials availability.

Reagents and strains generated from this study will be made available upon reasonable request with an appropriate materials transfer agreement.

## Author contributions

ALH, LCH, JL, MG, and GDW designed, and analyzed the bacterial cultures, human fecal testing, and mouse studies. ALH, LCH, and JL performed the bacterial culture and mouse studies. ESF, JF, LSL, and GDW designed the bioreactor studies. ESF and JF performed the bioreactor studies. CT, VT, and KB performed the sequencing analysis. FH and ADP performed and analyzed the NMR metabolite quantification data. ALH, MG, and GDW wrote the manuscript.

## Supplementary Material

Supplemental data

Supporting data values

## Version 1 03/21/2024

In-Press Preview

## Version 2 05/01/2024

Electronic publication
