# Supplementary material for: Dietary carbohydrates regulate intestinal colonization and dissemination of Klebsiella pneumoniae
Source: J Clin Invest. 2024 Mar 21;134(9):e174726. doi: 10.1172/JCI174726 (PMC11060737; doi:10.1172/JCI174726)
Supplement: Supplemental data [file jci-134-174726-s045.pdf]

Supplemental Material

Supplemental Table 1. Resources.

REAGENT or RESOURCE	SOURCE	IDENTIFIER
**Bacterial strains**		
Klebsiella pneumoniae MGH 78578	ATCC	700721
K. pneumoniae Δurease	This study	NA
K. pneumoniae Δ*ntrC*	This study	NA
**Biological samples**		
Human fecal samples	FARMM study	NA
**Chemicals**		
Kanamycin Sulfate	Fisher	BP906
Vancomycin Hydrochloride	Fresenius Kabi	NDC 63323-314-61
Neomycin Trisulfate Salt Hydrate	Sigma	N6386
Hygromycin B	Corning	30-240-CR
Apramycin Sulfate	Alfa Aesar	J66616
Polyethylene Glycol 3350	Miralax	NA
Dextran Sulfate, Sodium Salt	Fisher	BP1585
Lactulose	Thermo Scientific	J60160.22
10% Buffered Formalin Phosphate	Fisher	SF100-4
Phenotype Microarrays	Biolog	PM1, PM2A, PM3B
**Critical commercial assays**		
Quantichrom Urea Assay Kit	BioAssay Systems	76237-110
Hemoccult Assay	Beckman Coulter	60151
**Deposited data**		
FARMM Shotgun Metagenomics	FARMM study (33)	PRJNA675301

Experimental models: Organisms/strains		
C57Bl/6J	Jackson Laboratory	NA
**Plasmids**		
pMDIAI	Addgene	51655
pFLP-hyg	Huang,T *et al.* (67)	NA
pACBSR-hyg	Huang,T *et al.* (67)	NA
**Software and algorithms**		
Prism 9.5.1	GraphPad	NA

Supplemental Table 2. Oligonucleotides.

Name	Function	Sequence
Kp-NtrC-LRed_F	*ntrC* deletion Recombineering	GCCGGGTCATACCGAATTTTCGGTATACCTGCCTATTCGG AAGTAGAGGTGTTTatgCAAgggatccgtcgacctgcagttc
Kp-NtrC-LRed_R	*ntrC* deletion Recombineering	ACAAAATAGCAGCACTTTGCGCCGATCGGCTATTTTTCATC ATGCTGTTGAACCctaCTCatgtgtaggctggagctgcttc
Kp-UreaseOp-LRed-F	Urease operon deletion Recombineering	TACGGATGACATAAGCGTTTCGTATGACCGGGATAAACTC CCGCCGATCAATACTCATTGattccggggatccgtcgacc
Kp-UreaseOp-LRed-R	Urease operon deletion Recombineering	AGAGAGAGCAGAGGCTGCACCATCCGGACGCGCTTGCGC CCGGCTGGTGCAACAGGCCTAatgtgtaggctggagctgcttc
Kp-NtrC-Check-F	*ntrC* deletion test	GCACCGCTTTCCAGCTGACGC
Kp-NtrC-Check-R	*ntrC* deletion test	CCCGAATGCAGCAGTTCTCACGGG
Kp-UreaseOp-Check-F2	Urease operon deletion Test	CAATACGTTAGCAGCATGGAAAGGCAAAAGTTGC
Kp-UreaseOp-Check-R	Urease operon deletion Test	GCGGGGCGTAACGTAAGGTGTAATCT

Supplemental Table 3. Disease Activity Index.

Score	*Weight Loss (%)*	*Stool Consistency*	*Rectal Bleeding*
0	<1	Normal	None
1	1-5		
2	5-10	Loose Stool	Hemoccult Positive
3	10-20		
4	>20	Diarrhea	Gross Bleeding

Supplemental Table 4. DSS Inflammation Scoring System.

**DSS/Mucosal/Crypt Loss:**	Normal mucosa	0
	Shortening of basal one-third of crypts +/- slight inflammation and edema in lamina propria.	1
	Loss of basal two-thirds of crypts +/- moderate inflammation in lamina propria.	2
	Loss of all epithelium +/- severe inflammation in lamina propria +/- submucosa inflammation but with surface epithelium still remaining.	3
	Loss of all epithelium, including surface epithelium +/- severe inflammation in the lamina propria and submucosa +/- muscularis. An exudate containing cell debris, inflammatory cells, fibrin and mucus covers the damaged mucosa.	4
**Crypt Inflammation:**	Normal	0
	1-2 inflammatory cells	1
	Cryptitis	2
	Crypt abscess/dirty necrosis	3
**Lamina Propria Mononuclear Cells:**	Normal	0
	Slight increase	1
	Moderate increase	2
	Marked increase	3
**Neutrophils:**	Normal	0
	Slight increase	1
	Moderate increase	2
	Marked increase	3
**Epithelial hyperplasia:**	Normal	0
	Mild	1
	Moderate increase	2
	Discrete nest of regenerated crypts delineated from adjacent mucosa with no obvious disruption of overlying mucosal surface	3
**Edema/fibrosis:**	None	0
	Mild/focal/single layer of colon	1
	Moderate/multifocal/multiple layers	2
	Severe/widespread/transmural	3
**Maximum Total:**		19

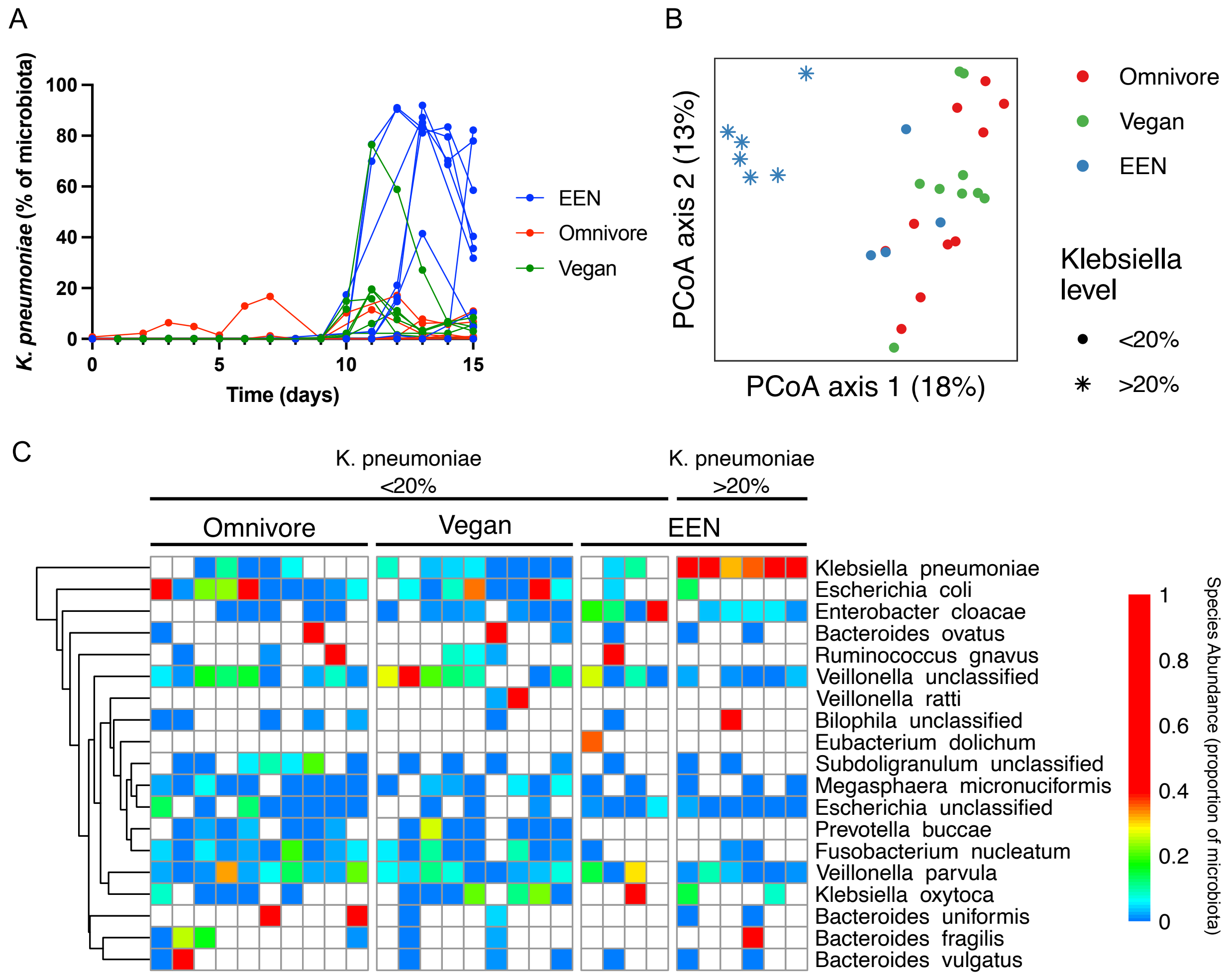

Supplemental Figure 1. A. Relative abundance of *K. pneumoniae* as a percentage of the microbiome determined via shotgun metagenomic sequencing, shown for each study subject, categorized by dietary group. This data is also presented in alternative graphical format in **Figure 1B**. B. PCoA plot of Bray-Curtis distances from each subject at Day 15 of the FARMM study. Dietary groups and *K. pneumoniae* high (>20%) and low (<20%) status are noted by color and symbol, respectively. **C.** Heat map of species abundance as determined by shotgun metagenomic sequencing of each subject, separated by dietary group and *K. pneumoniae* high or low categorization, from Day 15 of the FARMM study. n= 10 subjects per dietary group.

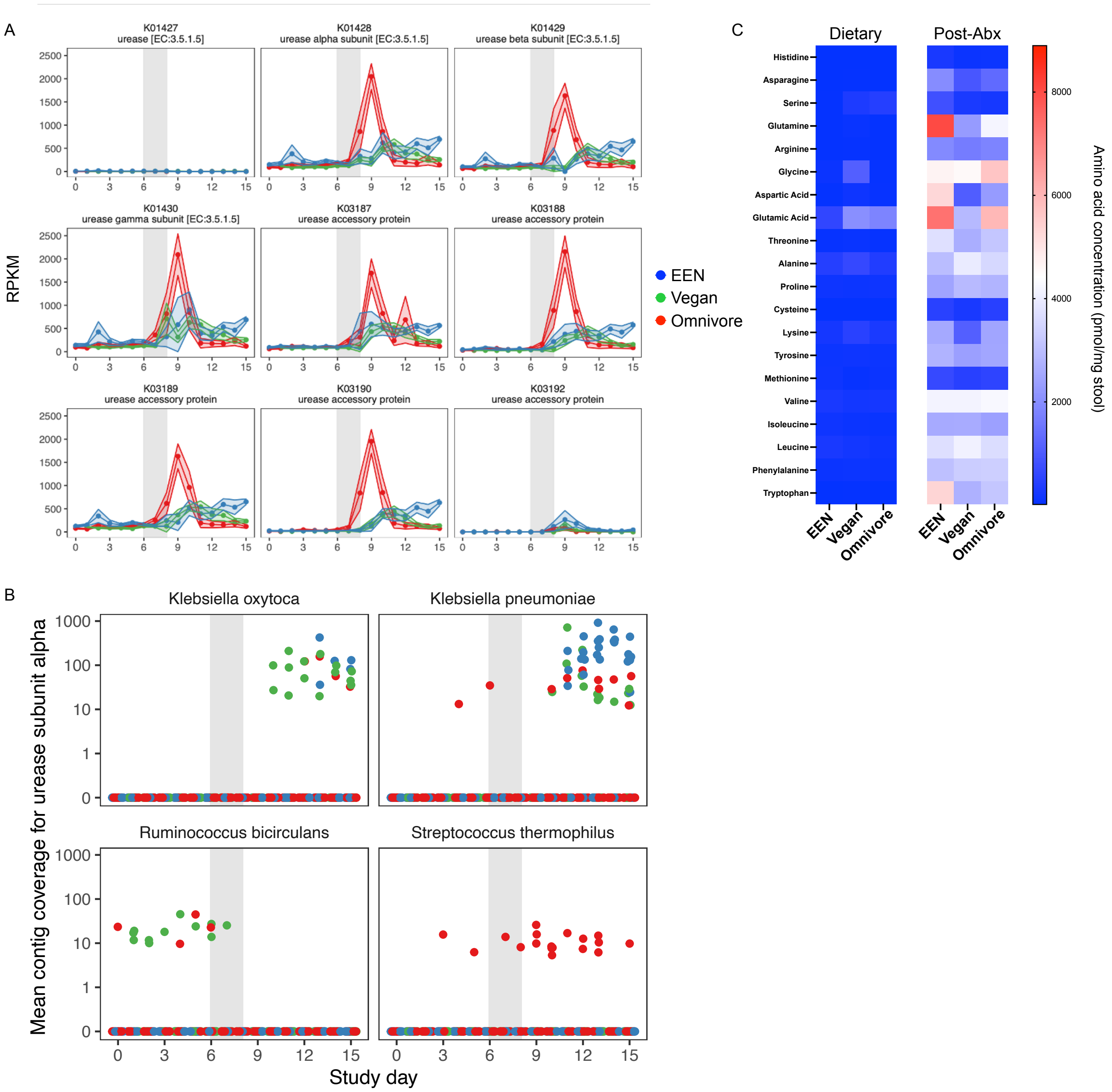

Supplemental Figure 2. A. Urease gene abundance corrected for gene length and sequencing depth determined from shotgun metagenomic sequencing, organized by diet and study day. **B**. Urease operon reconstruction from shotgun metagenomic sequencing, with contig coverage displayed by study day and diet (EEN blue, omnivore red, vegan green). **C.** Amino acid quantification from the FARMM study on days 5 (dietary) and 9 (post-abx), as determined by HPLC, organized by dietary group. Data displayed as mean ± SE (**A**). n=10 subjects per dietary group.

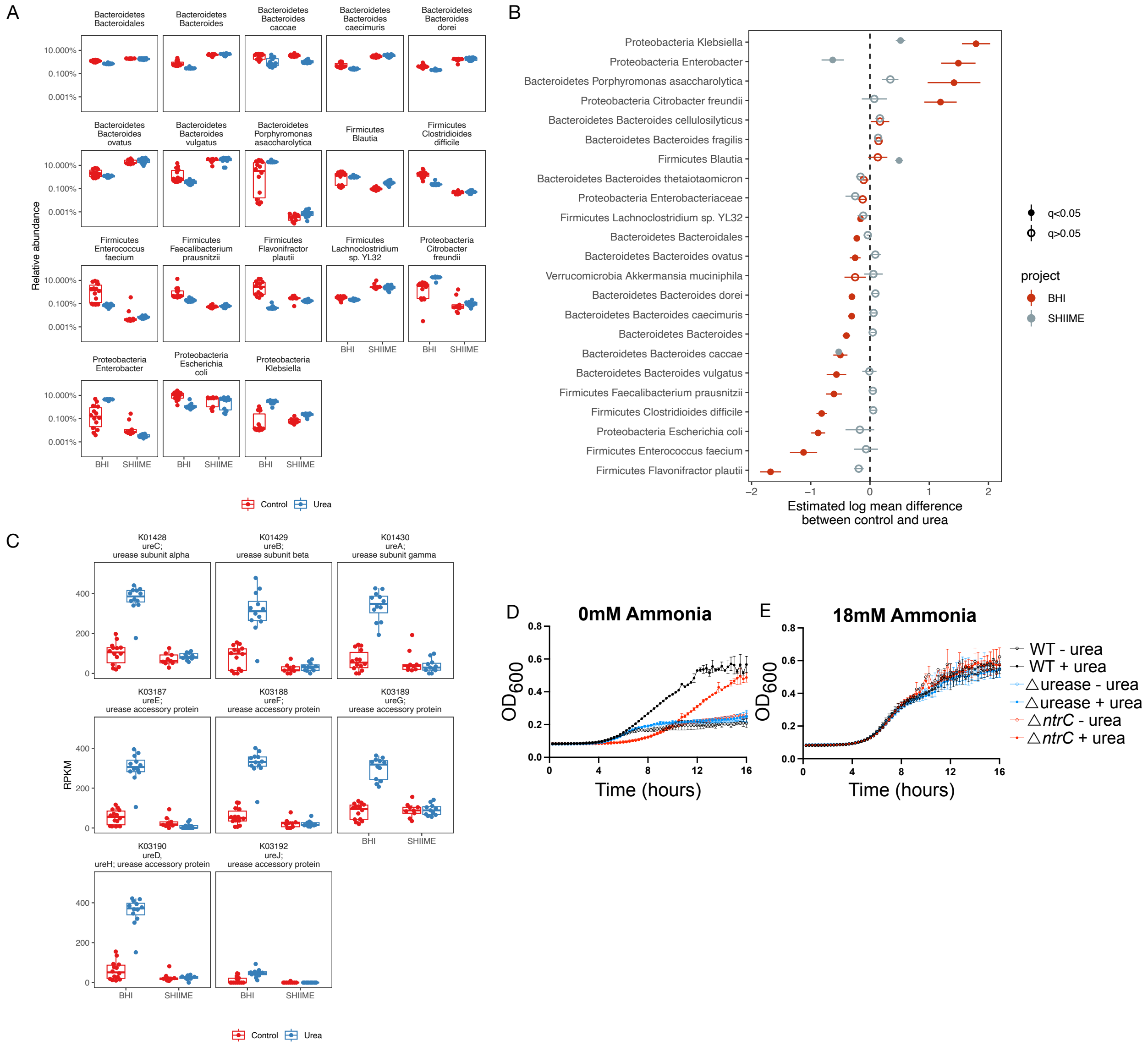

Supplemental Figure 3. **A-C**. A human fecal sample was inoculated into a bioreactor system with BHI media or SHIME media with or without urea supplementation. Serial samples were collected and subjected to shotgun metagenomic sequencing revealing differences in species abundance (**A-B**) and urease gene abundance (**C**). **D and E**. WT *K. pneumoniae* and isogenic mutants of the urease operon and *ntrC* gene grown in M9 minimal media with 0mM (**D**), 18mM (**E**) ammonia with or without 5mM urea supplementation. OD_{600} was monitored during aerobic growth for 16 hours. Data presented as mean with quartiles noted by box and whisker plot (**A and C**), median and first and third quartiles (**B**), or mean ± SD (**D and E**); n= 15 time points per condition (**A-C**), or n= 2-3 wells per condition (**D-E**). Data representative of three independent experiments (**D-E**). Results of linear model on log_{10} transformed abundance.

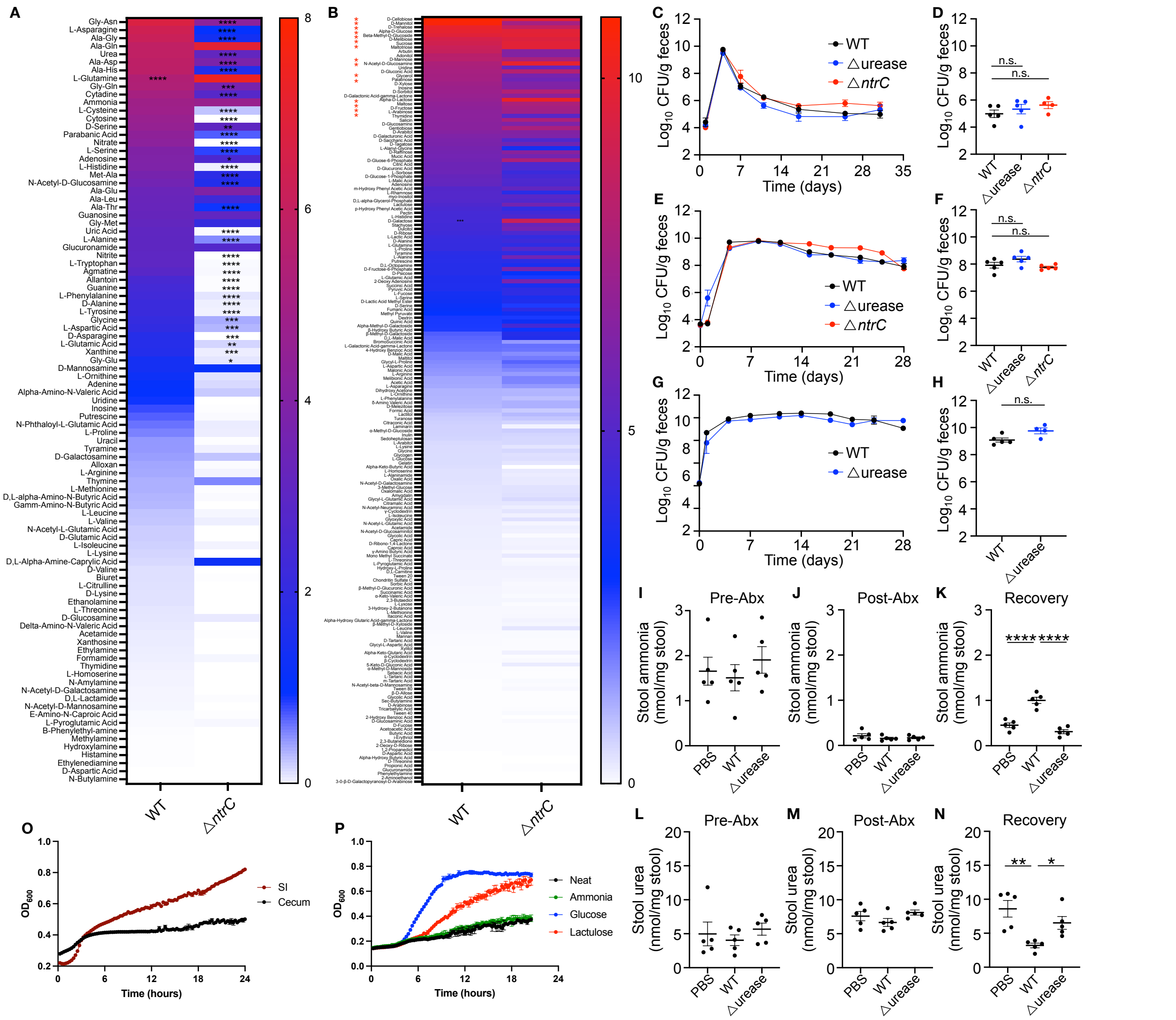

Supplemental Figure 4. A and B. WT and △*ntrC K. pneumoniae* were grown in M9 minimal media with single nitrogen sources (**A**) or carbon sources (**B**) as indicated. Heat map represents area under the curve of OD_{600} monitored during aerobic growth for 20 hours. Red asterisks denote simple or alcohol sugars in the top 25 listed compounds. **C-H**. Mice were provided a standard chow diet (**C and D**), a low protein diet (**E and F**), or a fiber free diet (**G and H**) and gavaged with WT, △*ntrC,* or △urease *K. pneumoniae* after abx pre-treatment and fecal CFU was monitored for the subsequent 4 weeks. (**C, E, and G**). CFU quantification 4 weeks after gavage for each respective diet (**D, F, and H**). **I-K**. Fecal ammonia was quantified in mice colonized with WT, △*ntrC,* or △urease *K. pneumoniae* before abx (**I**), after abx (**J**) and 3 days after gavage (**K**). **L-N**. Fecal urea was quantified in mice colonized with WT, △*ntrC,* or △urease *K. pneumoniae* before abx (**L**), after abx (**M**) and 3 days after gavage (**N**). **O and P**. *Ex-vivo* growth of *K. pneumoniae* in SI or cecal material (**O**) or in cecal material supplemented with ammonia, glucose, or lactulose (**P**) under anaerobic conditions. Data presented mean ± SEM (**C-P**), n=4-5 mice per group (**C-N**), or n=3 wells per experiment (**A, B, O, P**). Data represents two to three independent experiments. Results of one-way ANOVA with Bonferroni correction for multiple comparisons (**D, F, H, I-N**) or multiple t-tests with Bonferroni correction (**A, B**), n.s., not significant, n.s., not significant, *p<0.05, **p<0.01, ***p<0.001, ****p<0.0001.

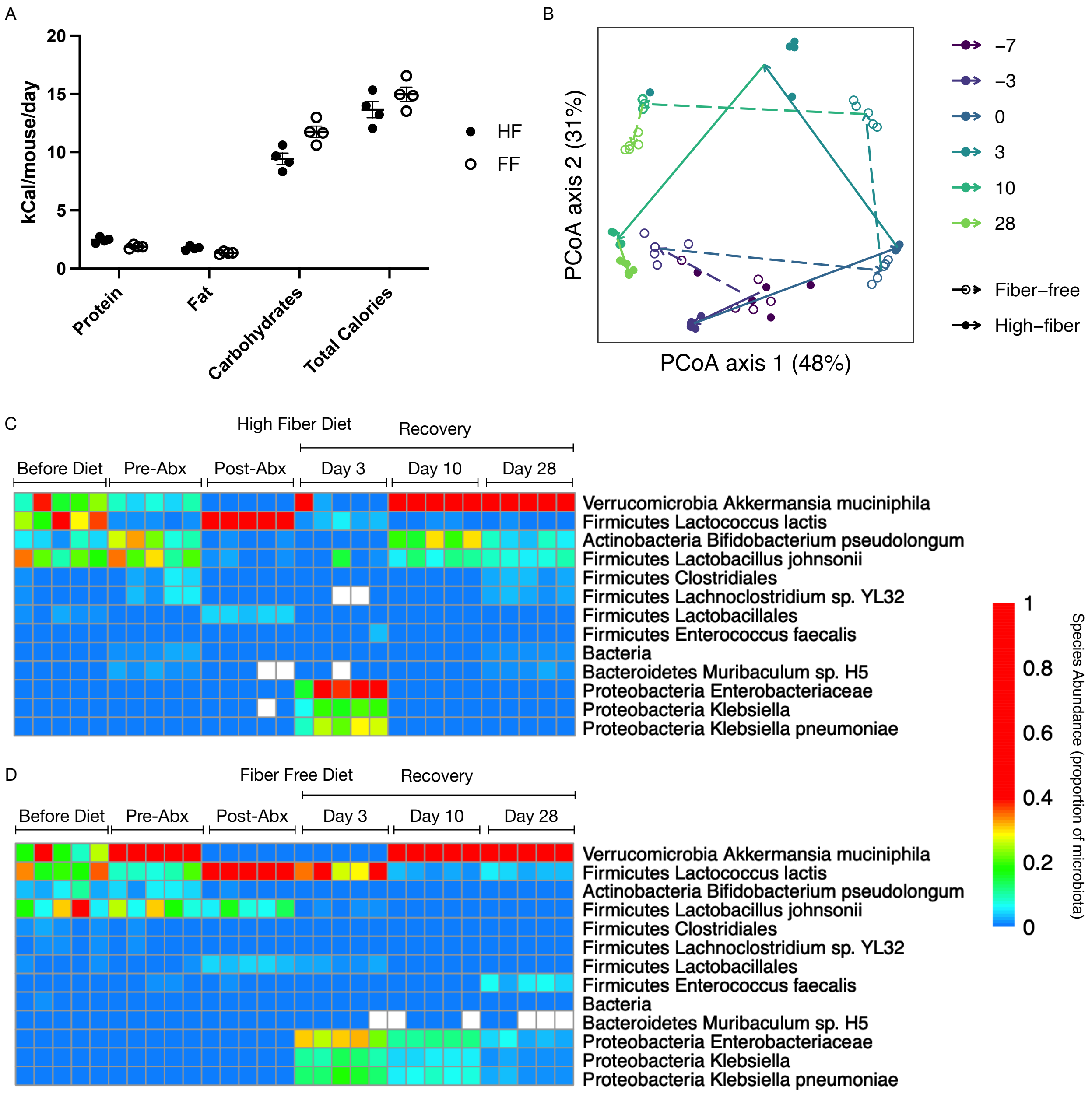

Supplemental Figure 5. A-D. Mice were provided a HF or FF diet, treated with antibiotics, and gavaged with *K. pneumoniae*. Calorie consumption of mice in HF and FF groups, organized by macronutrient (**A**). PCoA plot of Bray-Curtis distances from mouse shotgun metagenomic sequencing from HF and FF dietary groups over time, as denoted by color. Each mouse is indicated by a single point with lines and arrows connecting the centroids of consecutive timepoints of the study (**B**). Heat map of species abundance (**C and D**). Data presented as mean ± SD, n=5 mice per group. Average daily calorie consumption of mice in a group of 5 per cage, over a week period, each data point representing one cage (**A**). Result of multiple unpaired t-tests, FDR 1% (**A**).

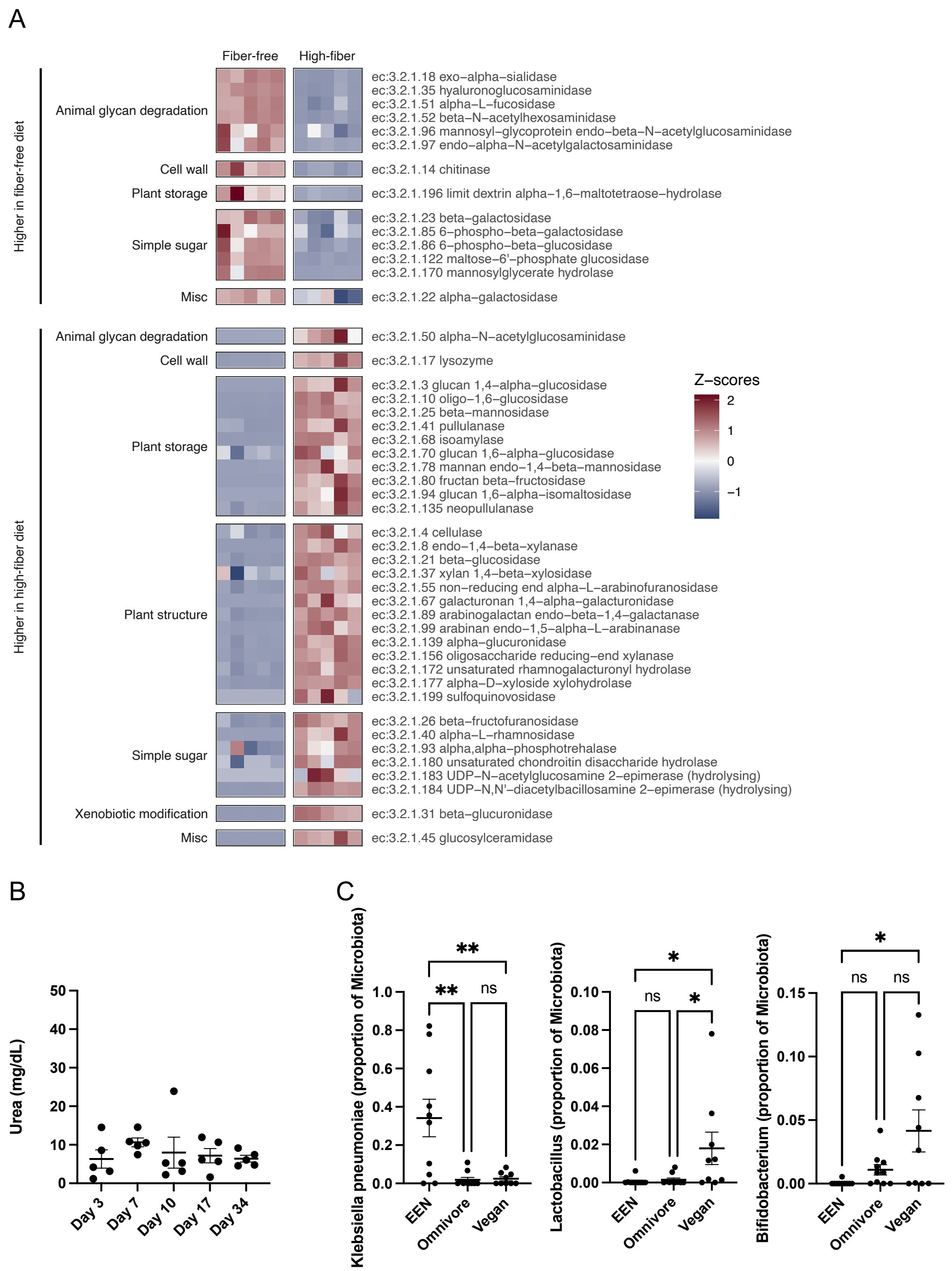

Supplemental Figure 6 A-B. Mice were provided a HF or FF diet, treated with antibiotics, and gavaged with *K. pneumoniae*. Z-scores of glycoside hydrolase abundance as determined by shotgun metagenomic sequencing over the course of the study period, with each mouse represented by a single column (**A**). Urea quantification from mice on a HF diet after *K. pneumoniae* gavage (**B**). **C.** Species abundance of *K. pneumoniae, Lactobacillus*, and *Bifidobacterium* from the FARMM trial shotgun metagenomic sequencing, organized by dietary group from day 15 of the study. Data presented as mean ± SD (**B and C**), n=5 mice per group (**A and B**), n=10 subjects per dietary group (**C**). The *K. pneumoniae* data of **C** is a different graphical format of the data from **Figure 1B**. Results of one-way ANOVA with Tukey correction, n.s. not significant, *p<0.05, **p<0.01 (**B and C**).
